# Periparturient Behavior and Physiology: Further Insight Into the Farrowing Process for Primiparous and Multiparous Sows

**DOI:** 10.3389/fvets.2018.00122

**Published:** 2018-06-12

**Authors:** Sarah H. Ison, Susan Jarvis, Sarah A. Hall, Cheryl J. Ashworth, Kenneth M. D. Rutherford

**Affiliations:** ^1^Animal Behaviour and Welfare, Animal and Veterinary Sciences, Scotland's Rural College, Edinburgh, United Kingdom; ^2^Royal (Dick) School of Veterinary Studies, University of Edinburgh, Roslin, United Kingdom; ^3^The Roslin Institute and Royal (Dick) School of Veterinary Studies, University of Edinburgh, Roslin, United Kingdom

**Keywords:** behavior, parturition stress, physiology, pain assessment, sow, welfare

## Abstract

Giving birth is a critical time for many species and is often the most painful event ever experienced by females. In domestic species, like the pig, pain associated with parturition represents a potential welfare concern, and the consequences of pain can cause economic losses (e.g., by indirectly contributing to piglet mortality as pain could slow post-farrowing recovery, reduce food and water intake, reducing milk let-down). This study investigated pain assessment and its management in primiparous (gilts) and multiparous (sows) breeding pigs, including the provision of a non-steroidal anti-inflammatory drug (NSAID) post-parturition. Individuals were randomly allocated to receive the NSAID ketoprofen (3 mg/kg bodyweight) (*n* = 11 gilts, 16 sows) or the equivalent volume of saline (*n* = 13 gilts, 16 sows) by intramuscular injection 1.5 h after the birth of the last piglet. Data collected included putative behavioral indicators of pain (back leg forward, tremble, back arch), salivary cortisol concentrations pre-farrowing and up to 7 days post-injection. In addition, post-partum biomarkers of inflammation, including the acute phase protein C-reactive protein (CRP) and 3 porcine cytokines [interleukin-1 β (IL1 β), interleukin-6 (IL6), and tumor necrosis factor α (TNF α)] were measured in plasma collected 6 h following the injection. Behaviors were analyzed using generalized linear mixed models, and physiological variables with linear mixed models. No difference in putative pain behaviors, salivary cortisol, CRP, or cytokines were found between individuals treated with ketoprofen or those administered the saline control. However, there were some differences between gilts and sows, as sows exhibited more putative pain behavior than gilts, had higher salivary cortisol on the day of farrowing and had higher plasma TNF α. Conversely, gilts had higher salivary cortisol than sows on day 3 post-farrowing and had higher CRP. This indicates that, like human females, multiparous sows experience more pain from uterine activity following birth than primiparas. This study provides useful information for developing management practices relating to post-farrowing care for breeding pigs.

## Introduction

For human females, giving birth is often the most painful event ever experienced, with a high percentage of women reporting severe or extremely severe pain (1). It is now widely accepted that many animals are capable of experiencing pain (2) and pain associated with parturition in domestic pigs has been discussed in a recent review (3).

Farrowing is a critical time in pig production, as success at this stage of the system means more piglets are weaned and ultimately sold. The breeding sow is essential for successful production around farrowing, and good health and welfare of the sow is reflected in her ability to produce a healthy litter of piglets. Any farrowing difficulties or reduced milk production can result in higher pre-weaning piglet losses (4). Parturition is likely to be painful in the pig as it is in other species as pain is likely to originate from uterine contractions, piglet expulsion and inflammation of the uterine tract from delivering a litter of piglets (3). After birth, pain in human females can be present from intermittent uterine contractions during the process of involution, when the uterus returns to its normal size and from tissue damage associated with a natural birth (5). Post-birth pain experienced by the mother has implications for her ability to recover in order to return normal activities and feed and care for her baby (5). It has been suggested that pain experienced by the sow post-farrowing, could affect her ability to feed and care for piglets (3, 4), which is a welfare concern for the sow and piglets and also an economic one for the farmer. This has resulted in recent research administering non-steroidal anti-inflammatory drugs (NSAIDs) post-farrowing and measuring the benefits to health, welfare and productivity (6–11).

Benefits of the post-farrowing administration of NSAIDs include a reduction in piglet mortality with the use of ketoprofen (6, 8), and an increased average daily weight gain of low birth weight piglets (< 1180 g) with the use of intramuscular meloxicam (7). A study in which oral meloxicam was administered as soon as possible after the onset of farrowing showed improved piglet weaning weight, average daily gain and evidence for improved immunoglobulin-G (IgG) transfer (12). Sows treated with intramuscular meloxicam spent less time lying on the third day following parturition (7) and younger sows treated with ketoprofen were more active than those given a placebo (11), which may indicate improved recovery post-farrowing. Other sow health and welfare benefits of ketoprofen included a reduced loss in back-fat and body condition score, a lower incidence of constipation and less severe shoulder sores (10). The administration of NSAIDs, in addition to antibiotics, has also been shown to aid in treatment of infectious causes of post-partum dysgalactia syndrome (PPDS) [e.g., (13, 14)] and, on farms with a high incidence of subclinical forms of the condition, piglet mortality was reduced (8).

Ketoprofen is an NSAID with anti-inflammatory, analgesia, and antipyretic properties, with a number of brands available and licensed to treat conditions involving pain, inflammation, and fever in pigs in the UK (15). It is absorbed well, reaching maximum levels after ~1 h following intra-muscular (IM) injection (16). Nociceptive thresholds were reduced in piglets with kaolin-induced inflammation, when administered ketoprofen compared with a placebo up to 24 h following IM injection (17). It is the second most popular NSAID in the UK, after meloxicam; reported to be used or prescribed by 50% of pig veterinarians (18). Ketoprofen is licensed for use in pigs in the UK to be used along with antimicrobials to treat respiratory disease, mastitis, and metritis (or PPDS) at a dose rate of 3 mg per kg bodyweight (15). Scientific research has demonstrated the efficacy of ketoprofen in experimentally infected pigs (19, 20), to treat respiratory disease (21), and non-infectious lameness (22). As previously mentioned, the post-farrowing administration of ketoprofen has shown health, welfare, and production benefits (6, 10, 11), especially on farms with a high incidence of PPDS (8).

This study investigated the assessment and management of pain in primiparous (hereafter referred to as gilts) and multiparous (hereafter referred to as sows) breeding pigs, including the administration of the NSAID ketoprofen after farrowing. A previous publication, as part of the same study, presented the effect of post-farrowing ketoprofen on gilt and sow feed intake, nursing behavior, and piglet performance (23). This article focuses on behavioral and physiological measurements taken from gilts and sows as indicators of stress and pain, including putative behavioral indicators of pain identified previously (24). Putative behavioral indicators of pain were also measured in the hours preceding farrowing, to investigate the onset of potentially painful uterine contractions as farrowing approached.

## Materials and methods

This experiment was carried out under UK Home Office License, in compliance with EU Directive 2010/63/EU, and following approval from the SRUC Animal Welfare and Ethical Review Body (AWERB).

### Gilt and sow housing and husbandry

Thirty-two Large White × Landrace multiparous [sows; mean parity 4.63 ± 0.43 (SEM)] and 24 primiparous (gilts) breeding pigs were used in this study. The gilts in this study were acquired directly from a breeding company (The Camborough®, PIC, UK), whereas the sows were home bred from an older genetic line of the same breed. Experimental procedures were carried out at the SRUC pig research farm (Midlothian, UK), with gilts and sows farrowing in 9 batches between February and October 2014. Approximately 4 days before the expected farrowing date, gilts and sows were moved into individual conventional farrowing crates (1.8 × 0.5 m), with solid concrete flooring (1.8 × 1.5 m), a small slatted area at the back (0.5 × 0.5 m), and a water and feed trough at the front. A heated creep area (1.5 × 0.65 m) was accessible to piglets, positioned in front of the water and feed trough. Individuals were fed a standard pelleted lactation diet (16.4% crude protein, 6.8% crude oils and fats, 4.0% crude fiber, 5.8% crude ash, 13.8% moisture, 0.8% calcium, 0.94% lysine, 0.25% methionine, 0.51% phosphorus, and 0.22% sodium) twice a day at 0745 and 1530 and had continuous access to fresh water. Gilts and sows were fed based on a feed chart, which was adjusted slightly according to the size, body condition, and appetite of the individual (e.g., gilts were fed slightly less than sows and a lower body condition score was given slightly more feed) and increased gradually from the day of farrowing to weaning. Gilts and sows were cleaned out daily at the morning feed, when they were provided with 2 handfuls of fresh, long-stemmed straw. Additional straw was added and any manure removed at the afternoon feed in the days preceding farrowing, to provide adequate nest-building material and maintain hygiene for the newborn piglets. Lights were switched on immediately before the morning feed, turned off at 1630 and an additional night-light was provided in the center of each room of crates.

Cross-fostering was conducted where necessary to even up litter sizes, in order to maximize piglet survival as per normal farm practice. Cross fostering was conducted independently of experimental treatments and was conducted only after the 6 h post-injection data collection point. When litter sizes were uneven, the largest piglet or piglets were removed and placed on a gilt or sow with a smaller litter. Piglets were given an intramuscular injection of iron on day 3 post-farrowing, as per normal farm practice. On the 4th week after farrowing [mean piglet age 26.39 ± 0.20 (SEM)], weaning took place, which involved moving gilts and sows out of the crates, followed by ear tagging and vaccination (CircoFLEX) of the piglets, which is normal farm practice.

### Blinding and drug treatments

This study was a randomized, blinded, placebo controlled trial, where gilts and sows were randomly allocated to receive a single intra-muscular (IM) injection of ketoprofen (Ketofen; Merial Animal Health Limited, Harlow, Essex, UK) or the equivalent volume of saline, 90 min following the birth of the last piglet. Ketoprofen-treated gilts and sows received 3 mg per kg bodyweight or 1 ml per 33 kg bodyweight rounded down to the nearest 0.5 ml (treated), and those that received the saline as a placebo control were given the equivalent volume (control). The 56 individuals were balanced as much as possible across the different batches and for parity between the 2 treatment groups (*gilts*: treated, *n* = 11, control, *n* = 13; *sows*: parity 2 to 4; treated, *n* = 9, control, *n* = 8; parity 5 to 7; treated, *n* = 5, control, *n* = 6; parity 8+; treated, *n* = 2, control, *n* = 2). After the first experimenter had allocated individuals to the 2 treatments groups, a second experimenter added the ketoprofen or saline dose to individual brown medicine bottles, sealed with rubber stoppers (Adelphi Healthcare Packaging, Haywards Heath, West Sussex, UK), which were labeled only with the individual gilt or sow ear tag identification number. Ketofen contains the active ingredient ketoprofen at 100 mg/ml contained in a solution of l arginine, benzyl alcohol (10 mg/ml), citric acid monohydrate, and water. It is a clear colorless solution, with low viscosity, making it indistinguishable from the saline placebo to the third experimenter who administered the injection, and collected the data, who was blind to the treatments.

In the days leading up to farrowing, individuals were closely monitored for signs of farrowing, which included observation at twice daily feeding, and through remote monitoring using a digital surveillance system. Once the piglet expulsion phase began, the time of each piglet birth was recorded and when a 90 min gap from the last piglet birth and the gilt or sow appeared to have finished farrowing, the ketoprofen or saline treatment was administered. Ketoprofen was injected into the neck muscle, just behind the ear using an 18 gauge, 1.5 inch needle attached to a PVC extension tube and using a 10 or 20 ml syringe (Henry Schein Animal Health, Dumfries, Dumfries and Galloway, UK). Individuals were then left undisturbed for 6 h, before sampling took place (see below). In case of any adverse effects of the drug being administered, and if any additional treatments were needed within 24 h (e.g., if gilts/sows showed signs of PPDS), the farm manager was given a list of the pig ear tags and treatments and was responsible for any additional treatments that were required, to avoid double dosing with NSAIDs.

### Gilt and sow behavior

Closed-circuit television (CCTV) cameras (LL20, infra-red cameras, FR concepts, Ireland) were mounted above each farrowing crate, and were connected to a computer to record behavior using GeoVision Digital Surveillance System software (ezCCTV ltd, Herts, UK). This surveillance system enabled remote monitoring. Digital video footage was collected and stored to be observed later using The Observer XT 11.0 (Noldus Information Technology, Wageningen, The Netherlands). Continuous behavioral observations were made for 5 min every hour for 24 h before the first piglet birth. Behavior recorded included gilt and sow posture and a set of other spontaneous putative pain behaviors described in Table 1. After farrowing, individuals were observed for 15 min every 1.5 h, starting 1 h after the last piglet was born for a total of 8 observations. After these 8 observations, 3 more 15 min observations were made at 3 h intervals. Therefore, the first 15 min observation was 0.5 h before the injection (Pre −0.5) and the remaining observations afterwards (Post 1.0, 2.5, 4.0, 5.5, 7.0, 8.5, 10.0, 13.0, 16.0, 19.0). Posture and putative pain behaviors were also observed during these post-farrowing observations (Table 1).

**Table 1:** Ethogram of behaviors observed during the study.

	**Behavior**	**Description**	**State**	**Event**
Posture	Stand	Upright, with all feet on floor	✓	
	Sit	Front legs straight and back end down on the floor	✓	
	Kneel	Front knees on the floor, with back legs straight	✓	
	Lie lateral	Lying on one side with udder exposed	✓	
	Lie ventral	Lying with the udder on the floor	✓	
Spontaneous behaviors	Tremble	Visible shaking as if shivering when in a lateral lying position	✓	
	Back leg forward	In a lateral lying position, the back leg is pulled forward and/or in toward the body	✓	
	Back arch	In a lateral lying position, one or both sets of legs become tense and are pushed away from the body and/or inwards toward the center, forming an arch in the back		✓
	Tail flick	Tail is moved rapidly up and down		✓
	Paw	In a lateral lying position, the front leg is scraped in a pawing motion		✓
	Piglet birth	A piglet is fully expelled from the sow		✓

*Behaviors were recorded as a duration in seconds (state) or frequencies/counts (event)*.

### Gilt and sow physiology

#### Salivary cortisol

Gilts and sows were saliva sampled after the morning (between 0845 and 0915) and afternoon (between 1545 and 1615) feed pre-farrowing, on the day of farrowing, including an additional sample 6 h after the injection, then up to 7 days after farrowing. Individuals were offered 2 large cotton buds (Millpledge Veterinary, Clarborough, Nottinghamshire, UK) on which to chew for ~30 s or until saturated with saliva. The cotton buds were placed into pre-labeled Salivette tubes (SARSTEDT AG & Co., Nümbrecht, Germany), which were sealed and centrifuged for 5 min at 1,400 g. The supernatant was pipetted into pre-labeled 1.5 ml tubes and stored at −20°C for assay at a later date. For each gilt and sow, samples from 2 days pre-farrowing, the day of farrowing, and days 1, 2, 3, 5, and 7 post-farrowing were assayed.

On the day of assay, saliva samples were removed from the freezer to thaw. All samples were centrifuged at 2,300 g for 5 min and the supernatant pipetted into a clean, pre-labeled 1.5 ml tube to remove any particulate matter in the sample. Using Coat-A-Count cortisol radioimmunoassay kits (Siemens Healthcare Diagnostics Ltd., Camberley, UK), 200 μl of standards, quality controls (QCs), and undiluted unknown saliva samples were pipetted in duplicate into the antibody coated assay tubes, followed by 1 ml of ^125^I tracer. A standard curve was placed at the beginning and end of the assay, followed by 3 QCs (low, medium, and high) and another set of QCs were added to the middle of the assay. Assay tubes were stored at 4°C overnight for incubation. On the following day, the tubes were emptied (apart from the total count tubes), counted with a gamma counter (LKB-Wallac 1261), and concentrations calculated using AssayZap software (Biosoft, Cambridge, UK). Salivary cortisol concentrations were reported as nanograms per milliliter (ng/ml) and the range of the assay was 0.125 to 50 ng/ml.

The lower and upper detectable limits for the salivary cortisol assays were 0.434 and 54.423 ng/ml respectively. The average intra-assay coefficient of variation (CV) across 7 assay runs was 5.92% (7.06, 6.67, 5.79, 4.96, 6.07, 5.43, and 5.44% for assay runs 1 to 7 respectively). The inter-assay CV, based on sets of 3 (low, medium and high) samples per assay run of known cortisol concentration was 10.56%.

#### C-reactive protein (CRP) and cytokines (IL1 β, il6, and TNF α)

At 7.5 h after the injection, a blood sample was collected from the dams. Due to a previous study, showing greater putative pain behavior in the first 6 h after the last piglet birth (24), all individuals were left as undisturbed as possible during this time. Therefore, at 6 h after the treatment/control injection, each gilt or sow had her tail and ear cleaned with antiseptic wipes. A topical local anesthetic cream (EMLA®) was then applied to the tail and ear, and cling film was placed around the tail over the cream and held in place with micro-pore tape, to form an occlusive dressing. To allow the anesthetic cream to take effect, all other samples and measurements were taken, and blood sampling took place ~1.5 h after the cream was applied. The tail vein was first attempted and if unsuccessful, the ear vein was used; it was possible to sample from the tail for 17 gilts and 27 sows and the ear vein was used for the remaining 7 gilts and 5 sows (gilts were tail docked, sows had intact tails). Firstly the tail vein was attempted, by holding the tail up, away from the body, feeling for the last moveable tail joint. At this point, on the midline of the tail, a 20 gauge, 1 inch vacutainer needle, attached to a vacutainer needle holder (Henry Schein Animal Health, Dumfries, Dumfries and Galloway, UK) was inserted at approximately a 45° angle. A 6 ml EDTA vacutainer (Henry Schein Animal Health, Dumfries, Dumfries and Galloway, UK) was pushed toward the needle to fill with blood and if the vein was not found, the needle was gently moved around until it was. If the tail vein was not successful after 3 attempts, the ear vein was used. Polypropylene tubing was wrapped around the ear and held in place using locking forceps, to allow the ear veins to become prominent. Once raised, a 21 gauge 0.75 inch winged vacutainer needle (Henry Schein Animal Health, Dumfries, Dumfries and Galloway, UK) attached to a vacutainer needle holder was inserted into the most prominent raised vein. The forceps were then removed and a 6 ml EDTA vacutainer was pushed onto the needle to collect blood, again, if blood was not drawn, the needle was gently moved until blood appeared in the vacutainer. Blood was immediately placed on ice, then moved straight into a refrigerated centrifuge (at 4°C) and centrifuged for 15 min at 1,400 g. Plasma was pipetted into 4 1.5 ml pre-labeled tubes and frozen at −80°C to be assayed at a later date.

Plasma samples were assayed for C-reactive protein (CRP) using a commercially available ELISA kit (Alpco ®, Salem, New Hampshire, USA), extensively used previously to measure levels of CRP in studies of pigs [e.g., (25)]. Samples were removed from the −80°C freezer the evening before the assay and placed at 4°C overnight to gradually defrost. On the day of the assay, samples were placed at room temperature for 30 min and were centrifuged for 1 min at 865 g before any further preparation took place. An initial test plate was run to establish the best dilution for the samples, with a 1:5,000 dilution chosen. Dilution buffer was prepared according to the instructions and samples were diluted using plain 96-well V-bottomed plates. A plate layout was created with standards, blanks and samples. According to the kit instructions, the CRP calibrator was reconstituted and the 6 standards created using a 2-fold serial dilution. Assay buffer was used for a zero standard and blanks. According to the plate layout, 130 μl of standards, samples and blanks were then pipetted into a plain V-bottomed plate, before 100 μl was pipetted into the antibody coated plate supplied with the kit. The assay was then conducted according to the manufacturer's instructions. Samples were assayed across 2 plates, with treatments and gilts and sows balanced across plates as much as possible and 3 samples and a reference serum that came with the kit, were run on both plates to act as quality controls (QCs) to calculate inter-plate variation. The plate was read using a Multiskan™ FC Microplate Photometer plate reader and results calculated using a 5 point logistic regression curve using Thermo Scientific SkanIt™ for Multiskan™ FC software (version 2.5.1) (Thermo Fisher Scientific Inc, Waltham, Massachusetts, USA). The assay range was 6.25 to 200 ng/ml and sample results with a CV% of greater than 10% were repeated.

Also using the gilt and sow plasma, simultaneous quantification of 3 porcine cytokines [interleukin-1 β (IL1 β), interleukin-6 (IL6), and tumor necrosis factor α (TNF α)] in a single plasma sample was conducted using multiplex fluorescent microsphere immunoassays (FMIA) with the BioPlex® 200 system (Bio-Rad Laboratories Ltd., Hemel Hempstead, UK). Details of the development, including optimization of this multiplex assay for porcine samples is currently in press (Hall et al., in preparation^1^) and further details for running the assay have also been published previously (27). Briefly, capture antibodies for each cytokine were coupled to microspheres (beads) using the Bio-Plex amine coupling kit following the manufacturer's instructions (Bio-Rad Laboratories Ltd., Hemel Hempstead, UK). Samples were removed from the −80°C freezer on the morning of the assay and put on ice before further processing. Samples were undiluted for the assay. Standards were created using a 2.5-fold serial dilution to create 7 standards from 10,000 to 40.96 pg/ml for each cytokine, Table 2 (adapted from Hall et al., in preparation^1^) shows the source of the recombinant protein for the standards and the capture and detection antibodies. To run the FMIA, firstly a mastermix solution of at least 3600 beads per region (cytokine) per well was produced, then 50 μl was added to each well of a black flat bottomed 96-well plate (BioRad catalogue #171025001). The plate was then washed twice with phosphate buffered saline (PBS) using a Bio-Plex Pro II wash station (Bio-Rad Laboratories Ltd., Hemel Hempstead, UK), with the magnetic plate washing carrier installed. Then, 50 μl of standards, unknown samples and blanks were added to the plate, which was incubated in the dark for 1.5 h at room temperature, with shaking at 700 rpm. Following this, the plate was washed 3 times (as before) and 75 μl of detection antibody was added and the plate was incubated for 40 min as before. After another 3 washes, 50 μl of Streptavidin-PE solution (Bio-Rad Laboratories Ltd., Hemel Hempstead, UK) was added and the plate was incubated for a further 20 min. The plate was then washed 3 times, 125 μl of assay buffer was added and the plate was incubated with shaking for 5 min. The reaction was measured using the BioPlex® 200 instrument and analyzed using the Bio-Plex manager software (version 6.1), using a 5 point logistic regression to calculate a standard curve for each cytokine. For each well, mean fluorescent intensity was analyzed for 100 beads for each cytokine. Samples were run across 3 plates and CV% of greater than 20% for any of the cytokines were repeated and if the CV failed to fall below 20%, the result was treated as a missing value.

**Table 2:** Capture and detection antibodies (Ab, with optimized concentrations), recombinant protein for standards and bead region used for each cytokine [nterleukin-1 β (IL1 β), interleukin-6 (IL6), and tumor necrosis factor α (TNF α)] in the multiplex assay.

**Analyte**	**Reagent**	**Catalogue #**	**Description/bead region**	**Optimized antibody concentration (μg/ml)/bead region**	**Source**
IL1β	Capture Ab	MAB6811	Mouse anti-porcine	20	RandD systems
	Detection Ab	BAF681	Biotinylated goat anti-porcine	0.5	RandD systems
	Standard	681-PI	Recombinant Porcine IL-1 β	–	RandD systems
	Bead	MC10029	Pro Magnetic COOH Beads 29	29	Bio-Rad
IL6	Capture Ab	AF686 BAF686	Goat anti-porcine	20	RandD systems
	Detection Ab	686-PI	Biotinylated goat anti-porcine	0.5	RandD systems
	Standard	MC10026	Recombinant Porcine IL-6	–	RandD systems
	Bead		Pro Magnetic COOH Beads 26	26	Bio-Rad
TNFα	Capture Ab	MAB690	Mouse anti-porcine	20	RandD systems
	Detection Ab	BAF690	Biotinylated goat anti-porcine	0.5	RandD systems
	Standard	690-PT	Recombinant Porcine TNF-α	–	RandD systems
	Bead	MC10055	Pro Magnetic COOH Beads 55	55	Bio-Rad

*Adapted from (Hall et al., in preparation). Ab, antibody; Source, R&D Systems, (Abingdon, UK), Bio-Rad, (Hemel Hempstead, UK)*.

The lower and upper detectable differences for the cytokine multiplex assay was 4.61 and 817.54 pg/ml for IL1 β, 0.23 and 1461.22 pg/ml for IL6, and 0.42 and 162.02 pg/ml for TNF α. The intra-assay CV% for IL1 β, IL6 and TNF α was 0.89, 2.89, and 6.13%, respectively and the inter-assay CV% was 6.40, 5.25, and 6.42% for IL1 β, IL6 and TNF α, respectively. For C-reactive protein, the intra-assay CV% was 1.77%.

### Data analysis

Unless stated at the start of each results section, data were available for all individuals. There were 11 gilts and 16 sows in the ketoprofen treated group (treated) and 13 gilts and 16 sows in the saline control group (control). An additional factor in this study was that 13 individuals, 5 gilts (4 treated and 1 control) and 8 sows (4 treated and 4 controls) required additional treatment in the days after farrowing for PPDS. Therefore, data were analyzed by treatment (treatment vs. control), parity group at the level of gilt vs. sow and whether additional treatment was needed (yes vs. no). All data were analyzed and descriptive statistics calculated using R version 3.3.3 (R core team, 2013). Data manipulation and summaries were conducted using the “dplyr” package and plotted using the “ggplot2” package. Results were considered statistically significant at *P* < 0.05 and tendencies will be discussed at *P* < 0.1.

Behavioral variables, including pre-farrowing and post-injection behaviors (Table 1) were analyzed using generalized linear mixed modeling, with the glmmPQL function in the “MASS” package. All models included farrowing batch and dam ID as random variables. Pre-farrowing behavior models included gilt or sow and time and their interactions as fixed factors. Post-injection behavior models included time, gilt or sow, additional treatment (yes or no), and treatment (treated or control) and their interactions as fixed factors. All models analyzing count data used the negative binomial family, and for duration variables, a two-step hurdle model was used (28). This was conducted by first analyzing the variables as a binomial outcome (if the behavior occurred or not: 1,0), then for the instances where the behavior did occur, the durations were analyzed with a gamma distribution and log link function.

Physiological variables were analyzed with linear mixed models, using the lmer function in the “lme4” package, with gilt/sow, treatment, additional treatment, and their interactions as fixed factors. Cytokines and CRP were analyzed with batch, and salivary cortisol with batch and dam ID in the random model. The salivary cortisol concentrations were log transformed to improve the model fit, and also included sampling time as a fixed factor, and *post-hoc* comparisons between fixed factors with day were made using the lsmeans function in the “lsmeans” package.

## Results

### Pre-farrowing behavior

**Figure 1:**
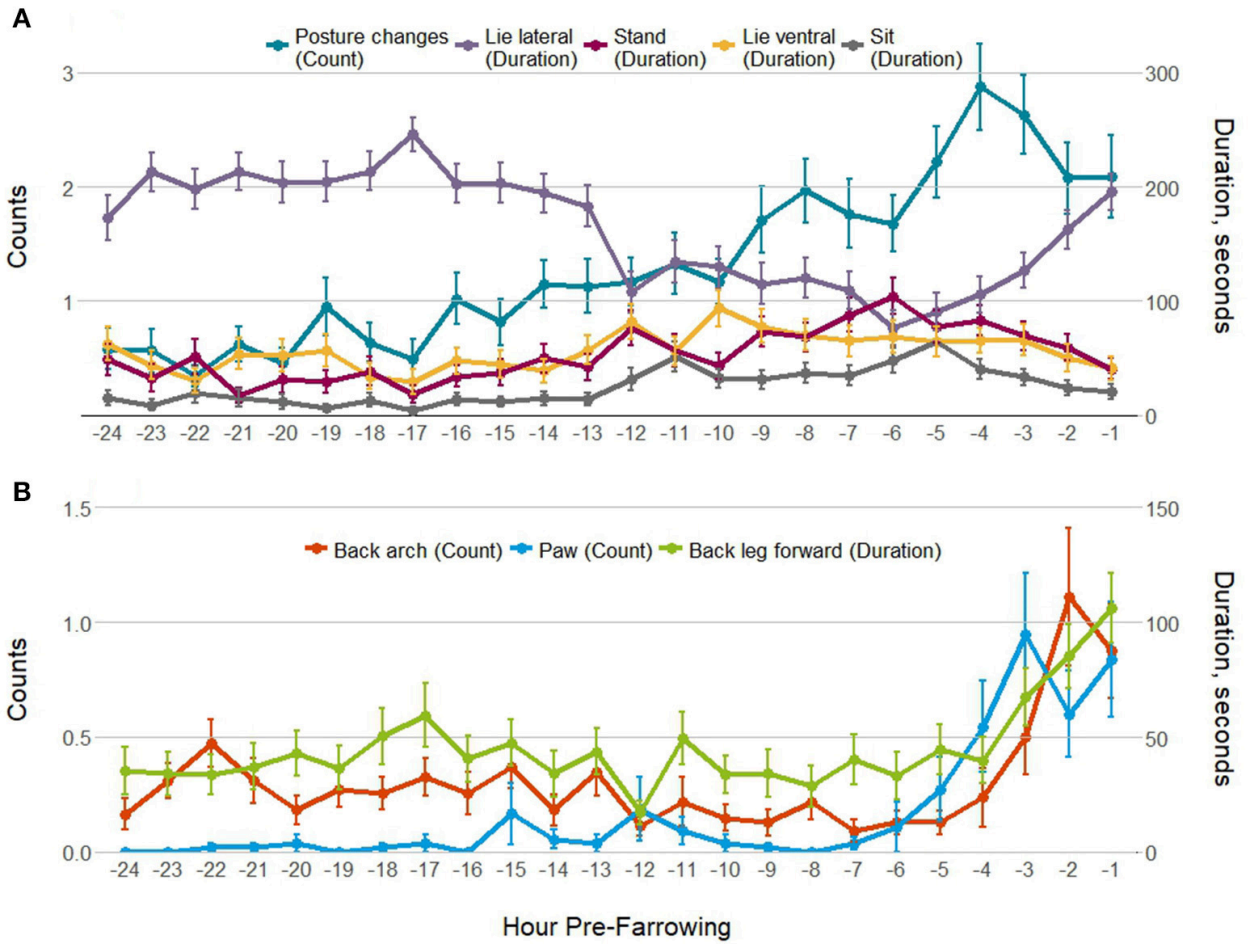
Mean ± SEM for **(A)** postures/posture changes; and **(B)** putative pain behaviors observed for 5 min every hour for the 24 h before the onset of farrowing.

Pre-farrowing behavior was observed for 55 of the 56 individuals; data were missing for 1 sow in the control treatment group. Tremble and tail flick were not analyzed pre-farrowing as these behaviors were rare. Figure 1 shows gilt and sow postures (Figure 1A), and putative pain indicators (Figure 1B) by hour pre-farrowing. All behaviors apart from sit differed by hour pre-farrowing (Table 3). Lying lateral decreased from 12 h pre-farrowing, whilst all other postures and the frequency of posture changes increased. In the last few hours before the onset of farrowing, lying lateral increased again, while other postures decreased, but the frequency of posture changes remained high, indicating restlessness (Figure 1A). In line with this restlessness, all putative pain behaviors followed a similar pattern: increasing in the last few hours before the first piglet birth (Figure 1B). Table 3 also contains the pre-farrowing behaviors observed by parity group (gilt or sow), along with the gilt vs. sow and gilt/sow × hour pre-farrowing interactions. Lie lateral, back arch and the frequency of posture changes differed overall between gilts and sows (Table 3), with greater lateral lying and more back arches observed in sows compared with gilts, and more posture changes in gilts than sows. There was a different pattern of lying lateral, back arch and posture changes between gilts and sows across the 24 h pre-farrowing, with significant gilt/sow × time pre-farrowing interactions (Table 3).

**Table 3:** Pre-farrowing **(a)** putative pain behaviors and **(b)** postures and posture changes with mean ± SEM for gilts and sows, and showing the statistical results for gilt vs. sow, and the gilt/sow × hour interaction.

	**Gilts vs. sow**			**Hour pre-farrowing**	**Gilt/sow × hour**
	**Gilt**	**Sow**	**t, *P***	**t, *P***	**t, *P***
**(a) PUTATIVE PAIN INDICATORS**					
Back leg forward, seconds	46.17 ± 3.61	43.68 ± 2.80	−0.9, 0.4	2.5, 0.01	0.6, 0.6
Back arch, frequency	0.25 ± 0.03	0.35 ± 0.03	3.5, 0.001	3.6, < 0.001	−2.6, 0.009
Paw, frequency	0.15 ± 0.04	0.18 ± 0.03	0.3, 0.8	4.4, < 0.001	0.1, 0.9
**(b) POSTURE**					
Stand, seconds	62.84 ± 4.38	45.39 ± 3.06	−1.7, 0.1	−3.3, 0.001	0.9, 0.4
Sit, seconds	22.05 ± 2.31	27.20 ± 2.33	0.6, 0.6	−0.7, 0.5	−0.3, 0.7
Lie lateral, seconds	150.33 ± 5.67	174.11 ± 4.88	2.2, 0.03	−3.3, < 0.001	−2.7, 0.008
Lie ventral, seconds	62.64 ± 4.06	50.48 ± 3.53	1.0, 0.3	−3.4, < 0.001	−1.4, 0.1
Posture changes, frequency	1.44 ± 0.08	1.22 ± 0.07	−3.0, 0.005	7.1, < 0.001	2.7, 0.007

### Post-injection behavior

**Figure 2:**
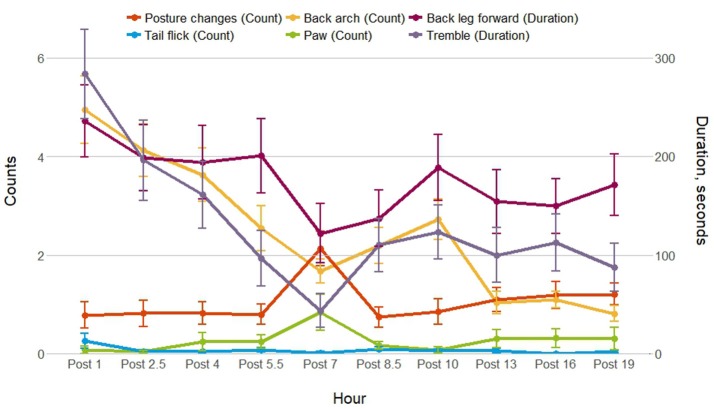
Mean ± SEM for posture changes, and putative pain behaviors observed for 15 min 1, 2.5, 4, 5.5, 7, 8.5, 10, 13, 16, and 19 h after the ketoprofen or saline injection.

**Figure 3:**
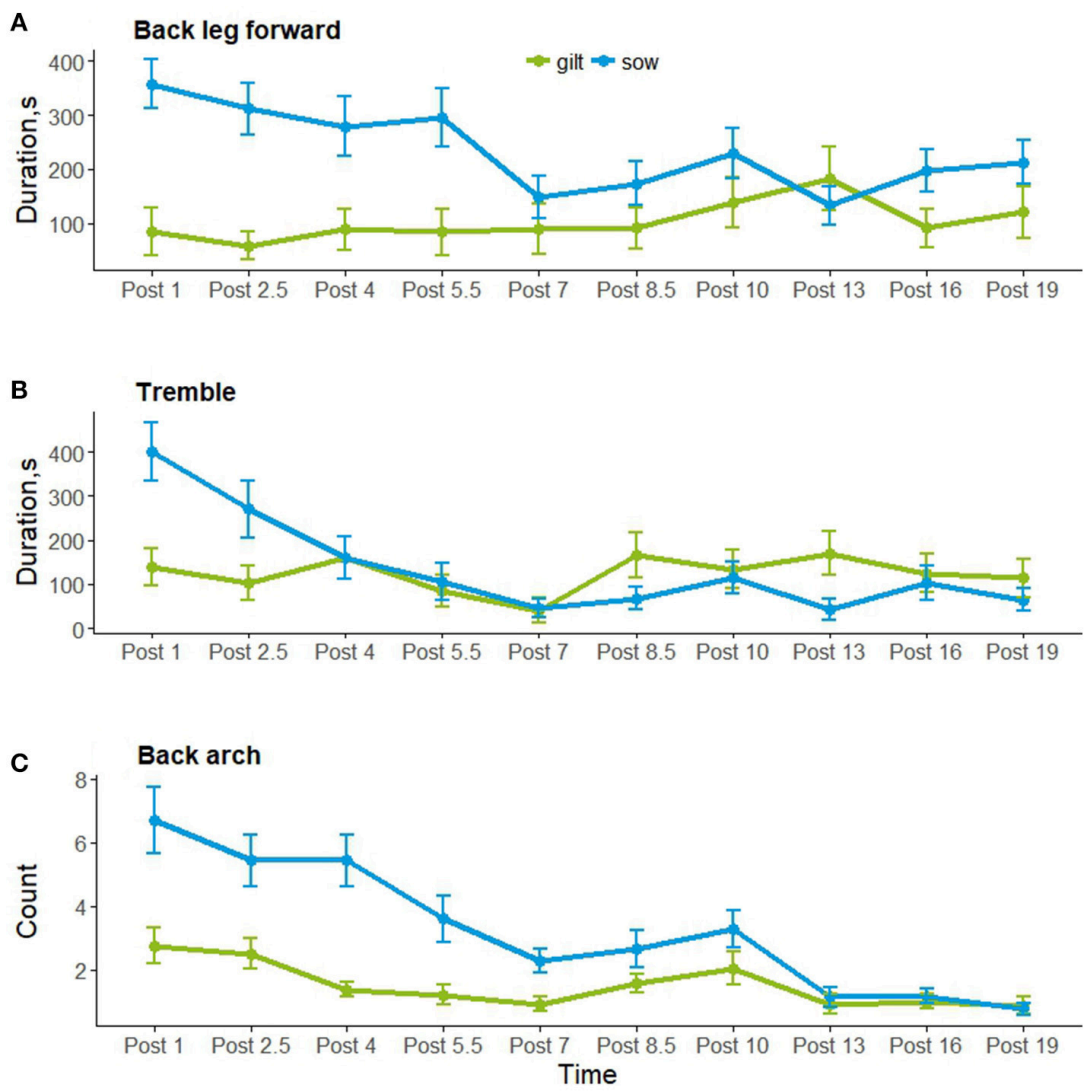
Putative pain behaviors (mean ± SEM) **(A)** Back leg forward; **(B)** Tremble; and **(C)** Back arch for gilts (in green) and sows (in blue) by hour post injection.

Post-injection behavior was observed for 54 of the 56 individuals; data were excluded for animals (1 control sow and 1 treated sow) who had more piglets after the injection was given. Figure 2 shows the frequency or duration of putative pain behaviors, and the frequency of posture changes observed by hour post-injection. As shown, back arch, tremble and back leg forward were lowest 7 h after the injection, as posture changes were highest, which coincided with the physiological data collection and piglet processing. Posture changes, paw and tail flick were not analyzed post-injection as these behaviors were infrequent. Back arch (*t* = −2.9, *P* = 0.004) differed by time, decreasing by hour post-injection, whereas back leg forward (*t* = 1.7, *P* = 0.09) and tremble (*t* = −0.7, *P* = 0.5) did not differ. Back leg forward, back arch and tremble did not differ by treatment, additional treatment or their interactions with time (Table 4). However, back leg forward and back arching differed between gilts and sows, along with significant gilt/sow × time interactions (Table 4; Figure 3). Gilts exhibited fewer back arches and less back leg forward behavior than sows, who began with high values, decreasing with hour post-injection, whereas gilts remained more stable across the observations.

**Table 4:** Overall duration or frequencies of putative pain behaviors for 10 15 min observations following the injection of a saline control or ketoprofen, with mean ± SEM displayed by treatment, for gilts and sows and by whether additional treatment was needed, along with effect size (t) and *P* values overall and for the interactions with time.

**Behavior**	**Treatment (treated vs. control)**			**Treated/ control × time**
	**Treated**	**Control**	**t, *P***	**t, *P***
Back leg forward, seconds	177.2 ± 15.0	173.5 ± 14.6	0.1, 0.9	−1.0, 0.3
Tremble, seconds	116.0 ± 13.7	145.8 ± 14.7	−1.6, 0.1	1.1, 0.3
Back arch, counts	2.5 ± 0.2	2.5 ± 0.2	0.3, 0.8	0.6, 0.5
	**Gilt/sow (gilt vs. sow)**			**Gilt/sow** × **time**
	**Gilt**	**Sow**	**t**, ***P***	**t**, ***P***
Back leg forward, seconds	102.6 ± 13.6	233.4 ± 14.6	2.3, 0.02	−2.1, 0.04
Tremble, seconds	123.7 ± 13.6	137.7 ± 14.6	1.4, 0.2	−1.3, 0.2
Back arch, counts	1.5 ± 0.1	3.3 ± 0.2	3.6, < 0.001	−2.4, 0.02
	**Additional treatment (yes vs. no)**			**Yes/no** × **time**
	**Yes**	**No**	**t**, ***P***	**t**, ***P***
Back leg forward, seconds	171.2 ± 21.1	176.3 ± 12.0	0.8, 0.4	0.1, 0.9
Tremble, seconds	108.0 ± 20.5	137.5 ± 11.6	0.4, 0.7	1.1, 0.3
Back arch, counts	2.9 ± 0.3	2.4 ± 0.2	0.8, 0.5	−0.5, 0.6

### Gilt and sow physiology

#### Salivary cortisol

**Figure 4:**
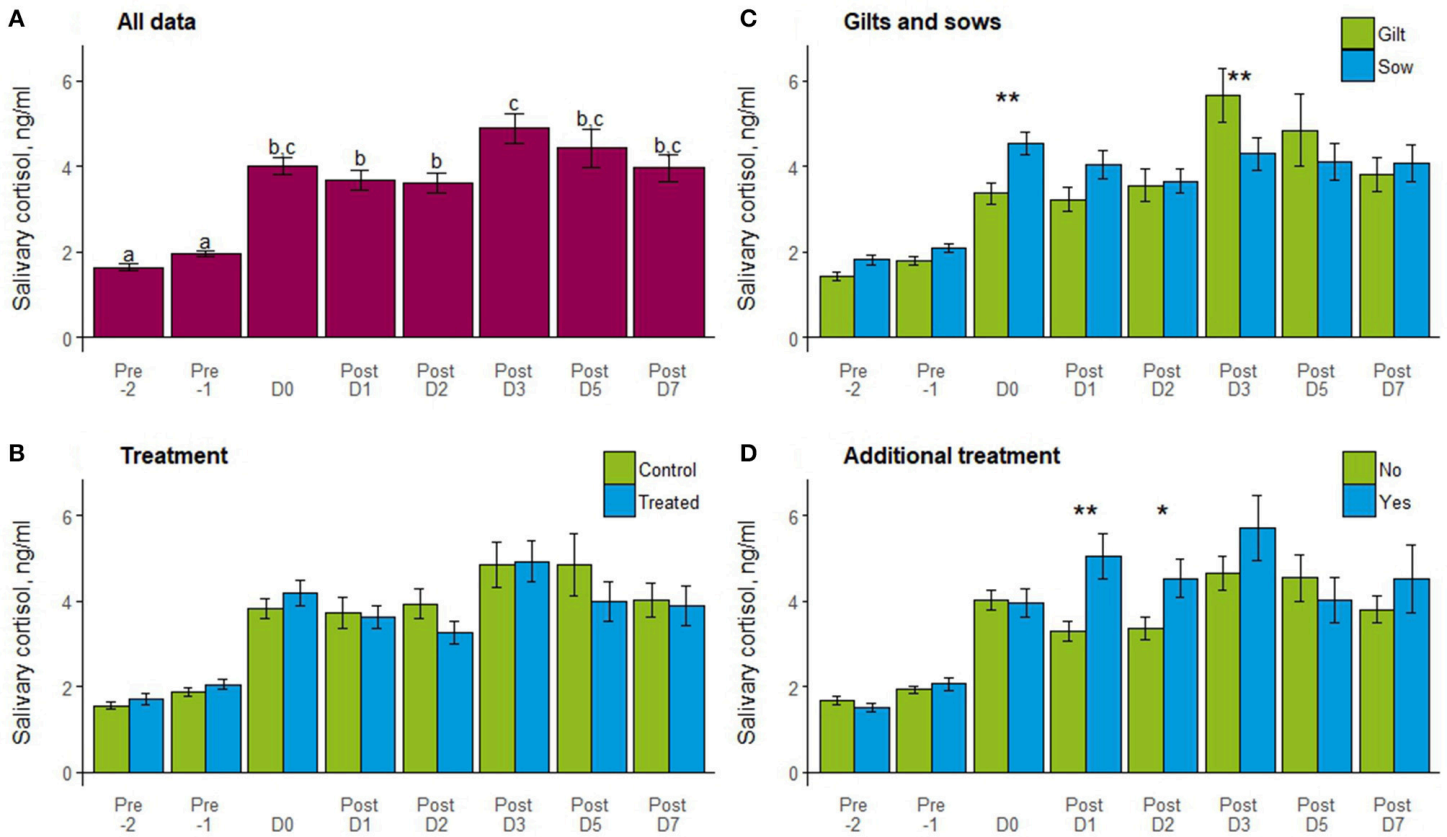
Mean ± SEM of salivary cortisol (ng/ml) by day in relation to farrowing (−2, −1, 0, 1, 2, 3, 5, and 7) for **(A)** all data; **(B)** treatments (control or treated); **(C)** gilts and sows and; **(D)** additional treatment (no or yes). Days with different letters show a significant difference and bars with a ** indicates a difference (*P* < 0.05), and * a tendency (*P* < 0.1) by day for treatment, gilt/sow, or additional treatment.

One sow was not saliva sampled due to aggression toward the sampler, so data presented are for 24 gilts and 31 sows. Salivary cortisol concentrations differed by day, with the lowest levels detected 2 days before farrowing (−2), rising significantly on day −1 and even more on the day of farrowing (Figure 4A). Cortisol levels remained high up to day 7 post-farrowing, with the highest levels measured on day 3 post-farrowing. Cortisol did not differ overall by treatment (*t* = 0.8, *P* = 0.4) or between control and treated individuals on any sampling day (Figure 4B). Gilts and sows did not differ overall (*t* = 1.6, *P* = 0.1), but differed in salivary cortisol on day 0 (*t* = −2.3, *P* = 0.02) with higher concentrations for sows than gilts, and on day 3 with higher concentrations for gilts than sows (*t* = 2.4, *P* = 0.02; Figure 4C). Individual requiring additional treatment or not post-farrowing due to PPDS did not differ overall (*t* = −0.3, *P* = 0.8) by those that did not, but those requiring treatment showed significantly higher cortisol on days 1 (*t* = −3.0, *P* = 0.003) and 2 (*t* = −2.5, *P* = 0.01) post-farrowing (Figure 4D).

#### C-reactive protein (CRP) and cytokines (IL1 β, IL6, and TNF α)

Due to a CV% of greater than 20% data are missing for interleukin-1 β (IL1 β) for 6 individuals, interleukin-6 (IL6) for 9 individuals and tumor necrosis factor α (TNF α) for 11 individuals. There were no significant differences for treatment, or by additional treatment for any of the cytokines or CRP (Table 5), however, TNF α was higher in sows than gilts (*t* = 2.2, *P* = 0.04), and CRP higher in gilts than sows (*t* = −4.5, *P* < 0.001).

**Table 5:** Mean ± SEM for interleukin-1 β (IL1 β), interleukin-6 (IL6), and tumor necrosis factor α (TNF α) and c-reactive protein (CRP) in gilt/sow plasma by treatment (control or treated), by parity group (gilt or sow) and additional treatment (yes or no).

**Protein**	**Treatment**		**Gilt/sow**		**Additional treatment**	
	**Control**	**Treated**	**Gilt**	**Sow**	**Yes**	**No**
IL1β, pg/ml	198.4 ± 37.6	136.8 ± 34.1	187.3 ± 43.5	156.5 ± 31.8	179.1 ± 70.7	165.9 ± 26.7
IL6, pg/ml	260.9 ± 71.2	251.4 ± 64.0	171.1 ± 33.7	303.9 ± 70.3	136.0 ± 47.7	301.7 ± 61.1
TNFα, pg/ml	28.1 ± 9.2	24.2 ± 8.1	17.8 ± 8.3^a^	32.0 ± 8.4^b^	27.4 ± 17.3	25.7 ± 6.3
CRP, μg/ml	444.0 ± 40.1	489.8 ± 48.6	561.2 ± 36.5^a^	350.4 ± 36.9^b^	451.2 ± 58.8	468.3 ± 36.7

*Values with different superscript letters indicate a significant difference (P < 0.05)*.

## Discussion

Non-steroidal anti-inflammatory drugs reduce pain and inflammation by reducing prostaglandin synthesis through the inhibition of cyclo-oxygenase (COX) enzymes that increase following cell damage (29). Prostaglandins, however, are involved in the process of parturition, as well as inflammation, and prostaglandins have been shown to increase toward the onset of parturition in pigs, peaking at the time of birth (30). Due to the inhibition of prostaglandins, NSAIDs have been used in human medicine to delay parturition in cases of preterm birth (31). However, as these drugs cross the placenta, NSAID use can result in detrimental side effects for the fetus, where prostaglandins play an important role in developmental and physiological regulation (32). This has led to concern over using these drugs in livestock during parturition or immediately after fetal delivery when placental tissue is being passed. However, in the current study, there were no apparent adverse effects following, to the piglets or dams, of the administration of ketoprofen 1.5 h after the last piglet was born. Two sows had more piglets after the injection was given, 1 in the ketoprofen and 1 in the control group. In a previous study in cattle, where the NSAID ketoprofen was administered immediately following calving, treated dams tended to be less likely to retain fetal membranes than untreated controls (33), and a recent study administering oral meloxicam at the onset of farrowing reported no adverse effects and enhanced benefits (12). Therefore, it could be beneficial to administer an NSAID earlier, without detrimental side effects and additional benefits include the provision of a therapeutic dose to piglets via the sow's milk to treat pain and inflammation from routine management procedures in piglets (34).

### Behavior

Pre-farrowing behavioral observations of gilts and sows showed an increase in activity, including a reduction in lying lateral, and an increase in other postures and posture changes, indicative of nest building behavior (35). Similar to previous studies, this change in activity, as shown by a reduction in lateral lying, was apparent from 12 h before the onset of farrowing [e.g., (36, 37)], and was more pronounced for gilts, compared with multiparous sows (37, 38). The putative pain indicators back leg forward, back arch and paw began to increase between 3 and 4 h before the onset of farrowing. This coincides with a change from nest-building activity to passivity, the increase in myometrial electrical activity and increasing oxytocin concentrations before the onset of piglet expulsion (36, 39).

Overall, there were no treatment differences in behavior between ketoprofen and control treated individuals post-injection. This was similar to previous studies measuring posture and posture changes in the hours immediately after farrowing in relation to the administration of analgesics (7, 9, 11, 40). This means that it was not possible to validate the putative pain behaviors as indicators of pain post-farrowing with the use of ketoprofen as an analgesic. This drug, however, may not have provided adequate analgesia for the pain experienced in the early post-farrowing period, which is likely to include visceral pain from uterine contractions moving placental material along the birth canal and uterine involution as the uterus returns to its normal size, as well as inflammation from the process of parturition (5). In rodent studies that measured similar putative pain behaviors in the periparturient period, opioid analgesics reduced the expression of these behaviors, confirming them as indicators of pain (41–43), and opioid analgesics may be needed to confirm these behaviors as pain indicators in periparturient sows.

The earlier administration of an NSAID, during or even before farrowing may increase the effectiveness, by acting at peripheral and central sites before the onset of nociceptive input (44). The use of analgesia administered before surgery has been shown to reduce post-surgical pain, for example, the use of an NSAID administered before castration in piglets [e.g., 45). Earlier administration of oral meloxicam, given as soon as possible at the onset of farrowing, improved piglet outcomes (growth rate, weaning weight, and IgG transfer) (12), compared with studies in which NSAIDs were given after farrowing (7, 10, 23). This result may also be reflected in indicators of stress and pain in the sow. However, the administration of NSAIDs before or during farrowing has the potential to have adverse effects on the sow and piglets. As well as reducing pain and inflammation by reducing prostaglandin and thromboxane through the inhibition of cyclooxygenase (COX) enzymes (29), NSAIDs could also inhibit parturition, as prostaglandin increases toward the onset of farrowing and peaks at the time of birth (30). When the NSAID indomethacin was administered at the onset of nest-building in sows, nest-building behavior was reduced, and parturition tended to be delayed (46). In addition, NSAIDs can cross the placenta, which can result in detrimental side effects for the unborn fetus, where prostaglandins play an important role in developmental and physiological regulation (31). As discussed, the earlier administration of oral meloxicam demonstrated enhanced benefits, without detrimental side effects (12). Meloxicam is a selective COX-2 inhibitor (47), which could make it more suitable for the treatment of inflammation (48) when given earlier in the farrowing process than non-selective COX inhibitors, like ketoprofen (17). Therefore, it cannot be assumed that administering ketoprofen before or during parturition would have similar benefits to meloxicam.

Gilts and sows were disturbed around the sampling and piglet processing time, around 7 h after the injection, showing more posture changes and less putative pain behavior around this time. This behavior then increased slightly in the remaining post-injection observations. There were large overall differences in the expression of putative pain behavior between gilts and sows, with gilts showing less back arching and back leg forward behavior post-injection than sows, and with a different profile of behavioral expression across the observations. This is a paradoxical result as primiparous dams are generally considered to experience more pain during the fetal delivery phase of parturition than multiparous dams (3, 49). In addition, farmers considered pain at farrowing more often a problem for gilts than sows, and that a greater percentage of gilts than sows have difficulty farrowing (50). It could be that inexperienced gilts are more reluctant to show signs of pain or exhibit pain in a different way to more experienced sows. Alternatively, it is reported in human females that uterine contractions post-birth, during the process of uterine involution as the uterus contracts and returns to its pre-gestation size, is more painful for multiparous, compared with primiparous women due to the loss in uterine tone (5). These after-birth pains are enhanced during breast feeding as oxytocin is released causing the uterus to contract, and the pain reported by human females increases with parity, as well as an increase in the frequency and intensity of uterine contractions recorded using tocodynamometry (51). This could explain why more back arching and back leg forward behavior was seen post-farrowing in sows compared with gilts, as it is likely that these putative pain indicators are from pain due to uterine contraction (24). The authors of the human study suggested childbirth could have induced central neural changes that increased the predisposition for pain during the post-partum period (51). Another explanation for the differences in putative behavioral indicators of pain could be learned pain from previous experiences of parturition. In humans, fear of pain is thought to contribute to labor-related anxiety, since anxiety and pain are highly correlated (52). Excessive anxiety can lead to increased catecholamine secretion that may enhance nociceptive stimuli from the pelvis and increase the perception of these stimuli at the cortical level (52). Pain has been shown to be under the constant influence of learning processes affecting the anticipation of, and responses to future pain, as temporary periods of conditioned hyperalgesia were induced by both the predictability and uncertainty of pain from an electric shock (53). This has interesting implications for the perception of farrowing pain in primiparous and multiparous sows. The farrowing environment can be stressful, with different noises, odors, and often includes confinement to a farrowing crate. This could contribute to anxiety due to novelty in inexperienced gilts, which could enhance the perception of pain, or be associated with a painful event for experienced sows. Combining an already stressful environment, with fear of pain for experienced sows could be contributing to an increase in putative behavioral indicators of pain.

### Salivary cortisol

For all gilts and sows, salivary cortisol concentrations increased from 2 days before farrowing, to 1 day before farrowing and doubled on the day of farrowing. This is similar to previous studies where salivary (54) and plasma (37, 55–57) cortisol were also shown to increase in the days leading up to and the day of farrowing. In this study, salivary cortisol concentration remained high up to day 7 post-farrowing, which is in contrast to previous studies where plasma cortisol concentrations returned to pre-farrowing levels 2 days after farrowing in one study (57) and another study, but only for sows housed in free-farrowing pens (54). However, similar to the current study, sows housed in crates have previously been shown to have elevated salivary cortisol concentrations for at least the 5 days post-farrowing when cortisol was sampled (54). Therefore, confining sows to a farrowing crate could be contributing to elevated salivary cortisol during this time.

Gilts and sows showed different salivary cortisol profiles; in line with the behavioral results, sows had higher cortisol on the day of farrowing, but gilts had higher concentrations on day 3 post-farrowing. This is an interesting result as previous research has shown a greater increase in cortisol in the pre-farrowing nest building phase in crated compared with penned gilts (55), but a smaller difference was seen in sows (37). Therefore, a greater pre-farrowing and farrowing day cortisol concentration was expected for the gilts, compared with the sows in this study. However, in the previous studies, samples were taken in relation to farrowing, whereas in this study, samples were collected at husbandry times, so may not have picked up the more detailed alterations in cortisol profiles with nest building and leading up to farrowing. However, it was important to measure salivary cortisol at the same time each day due to diurnal variations in the concentrations of this hormone, as well as limit the behavioral disturbance caused by saliva sampling. An increase in cortisol is not necessarily an indication of psychological stress, it also indicates the involvement of the metabolic process, including the mobilization of energy to create homeostasis (58). Nest-building activity, farrowing and the onset of lactation is physically demanding, resulting in an increased energy requirement, which could, in part, explain the increase in salivary cortisol concentrations, especially considering the feed restrictions imposed on the modern domestic sow. Therefore, the different salivary cortisol profiles shown by gilts and sows could reflect different metabolic demands associated with farrowing and lactation between these 2 groups of animals. It should be noted, however, that a 24 h feed restriction treatment on grower pigs failed to elicit a salivary cortisol response (59). Therefore, another explanation for the gilt/sow difference in salivary cortisol could be from individual genetics, as the gilts were obtained directly from a breeding company, whereas, the sows were home-bred from older genetic stock.

Higher concentrations of salivary cortisol on days 1 and 2 post-farrowing was measured in individuals that required additional treatment compared to healthy animals. It is possible that as these animals consumed less feed, they may have needed to mobilize energy reserves and increased cortisol could be an indicator of this. In addition, it could also be that being unwell is stressful and unpleasant, causing an increased cortisol concentration. In a study where plasma cortisol was measured in sows experimentally inoculated with *Escherichia coli* into the mammary gland on the day of farrowing, 4 out of 12 sows in the inoculated group developed clinical signs of mastitis (60). All sows showed an increase in cortisol associated with farrowing, but the 4 sows that developed clinical signs of mastitis had a greater cortisol increase. Another study showed greater plasma cortisol levels in sows with mastitis-metritis-agalactia (MMA), compared to healthy sows on days 1, 5, 10, 15, and 20 post-farrowing (61). In order to better evaluate stress responses in pigs, measuring a variety of salivary biomarkers is recommended, including indicators of the sympathetic adrenomedullary system, as well as the hypothalamic–pituitary–adrenocortical axis (59).

### C-reactive protein (CRP) and cytokines

To minimize disturbance in order to enhance the quality of behavioral data obtained during this study, only one blood sample was taken for analysis of CRP and cytokines. It should be noted that one sample, with no baseline, limits the interpretation of the CRP and cytokine results in this study. In addition, the impact of the blood sampling itself on plasma concentrations of these biomarkers cannot be discounted, despite efforts to minimize sampling stress by sampling from the tail vein, not using additional restraint (other than farrowing crate housing) and using a topical anesthetic. Despite limitations of interpretation, the results will be discussed in relation to previous studies measuring APPs and pro-inflammatory cytokines in the periparturient period.

Acute phase proteins (APPs) such as CRP, and pro-inflammatory cytokines are useful measures of inflammation and tissue damage, used to monitor, detect and diagnose disease (62). CRP has been identified as a good marker of inflammations in pigs, increasing 8-fold with experimentally induced sterile inflammation, peaking at 2 days after the injection of turpentine (63). Previous studies have shown an increase in APPs and cytokines in relation to farrowing, regardless of other treatments being studied, which could be an indication of tissue damage and inflammation associated with the farrowing process (10, 60, 64, 65). Neither gilt or sow CRP nor the cytokines IL1 β, IL6, or TNF α differed with ketoprofen treatment, which was similar to a previous study where APPs were measured in sows in relation to the administration of ketoprofen post-farrowing (10). In this previous study, the APPs haptoglobin (Hp) and serum SAA were measured on the day before, the days of, then 5 and 14 days post-farrowing, demonstrating that SAA, but not Hp was elevated in response to farrowing. On day 5, SAA was more elevated in the ketoprofen treated sows, compared with controls, which the authors suggested could be due to tissue irritation from the repeated ketoprofen injection (10), but no such result was seen with CRP in the current study. Although CRP was only measured at one time point in this study, due to high values obtained, in comparison to other studies (66–68), it is likely that CRP was elevated in response to parturition, but further samples would need to be taken to confirm this. It is also worth mentioning that, compared with the current study, 2 previous studies where IL6 and TNF α were measured using ELISA in parturient sows obtained higher values of TNF α and lower values of IL6 (60, 65) and another study showed similar values (69).

Primiparous sows had higher plasma concentrations of CRP than multiparous sows, and TNF α was greater for sows than gilts. In a previous study, parity influenced serum concentrations of Hp in that primiparous sows had higher values than multiparous sows (70). In contrast to salivary cortisol, no differences in plasma concentrations of CRP, IL1β, IL6, or TNFα were detected between sows requiring additional treatment, compared with those that did not. In a study where sows were experimentally inoculated with intra-mammary *E. coli*, elevated IL6 and TNF α was measured in inoculated sows 24 h post-inoculation, and was greater in sows that developed clinical signs of mastitis (60). Another study detected a difference in IL6 and TNF α in sows with MMA, compared with healthy controls at sampling points pre- and post-farrowing, as well as detecting an increase in these cytokines with parturition (65). It is possible that 6 h after farrowing may have been too early to detect a difference, as a previous study detected a difference at 48–72 h, but not 12–24 h after parturition (65) or that the degree of PPDS in the current study was not severe enough to create an acute phase response. Another study found no difference in CRP or Hp in sows with vulvar discharge syndrome (VDS), compared with healthy controls, although severe cases of VDS were not seen (67). It is important to note that previous studies involved clinical examinations of sows and confirmed the involvement of bacterial pathogens, which was not done in this study.

## Conclusion

In line with the previous paper, reporting the production results from the same study, which did not demonstrate clear benefits to the immediate post-farrowing administration of ketoprofen (23), results of the current study failed to show a change in indicators of pain, stress and inflammation with the use of post-farrowing ketoprofen. Further investigation on the timing and types of drug administration, including different regimes for primiparous and multiparous sows could enable more targeted use of drugs, with potential for improved sow and piglet outcomes. However, this study did highlight interesting differences between gilt and sow behavior and physiology, providing a novel insight into the farrowing experience for naïve and experienced individuals. It is generally thought that the piglet expulsion phase is likely to be more painful and/or problematic for gilts than sows, whereas the current study showed that pain in the immediate post-farrowing period could be greater for sows than gilts. This has implications for the management of farrowing sows, including the timing, and type of potential management interventions, including closer observation of experienced sows in the hours post-farrowing.

## Author contributions

SI was responsible for the experimental design, data collection and analysis, and manuscript preparation. KR provided significant supervision and advice at all stages of the study. CA and SJ provided advice on the experimental design and manuscript preparation, including useful input into the interpretation of results. SH conducted the cytokine assays and assisted with the manuscript preparation.

### Conflict of interest statement

The authors declare that the research was conducted in the absence of any commercial or financial relationships that could be construed as a potential conflict of interest.

## Footnotes

^1^Hall, S. A., Ison, S., Owles, C., Coe, J., Sandercock, D. A., and Zanella, A. J. (in preparation). Development of porcine Fluorescent Microsphere Immunoassay: Validation of assay conditions and sample treatment. *Methods X*.
